# An integrated analysis of miRNA and gene copy numbers in xenografts of Ewing's sarcoma

**DOI:** 10.1186/1756-9966-31-24

**Published:** 2012-03-20

**Authors:** Neda Mosakhani, Mohamed Guled, Gayle Leen, Silvia Calabuig-Fariñas, Tarja Niini, Isidro Machado, Suvi Savola, Katia Scotlandi, José Antonio López-Guerrero, Antonio Llombart-Bosch, Sakari Knuutila

**Affiliations:** 1 Department of Pathology, Haartman Institute and HUSLAB, University of Helsinki and Helsinki University Central Hospital, Helsinki, Finland; 2 Department of Information and Computer Science, Aalto University School of Science and Technology, Espoo, Finland; 3 Department of Pathology, Universitat de Valencia Studi General, Valencia, Spain; 4 Laboratorio di Ricerca Oncologica, Istituto Ortopedico Rizzoli, Bologna, Italy; 5 Laboratory of Molecular Biology, Fundación Instituto Valenciano de Oncología, Valencia, Spain

**Keywords:** Ewing's sarcoma xenograft, MicroRNA, Copy number, Microarray

## Abstract

**Background:**

Xenografts have been shown to provide a suitable source of tumor tissue for molecular analysis in the absence of primary tumor material. We utilized ES xenograft series for integrated microarray analyses to identify novel biomarkers.

**Method:**

Microarray technology (array comparative genomic hybridization (aCGH) and micro RNA arrays) was used to screen and identify copy number changes and differentially expressed miRNAs of 34 and 14 passages, respectively. Incubated cells used for xenografting (Passage 0) were considered to represent the primary tumor. Four important differentially expressed miRNAs (miR-31, miR-31*, miR-145, miR-106) were selected for further validation by real time polymerase chain reaction (RT-PCR). Integrated analysis of aCGH and miRNA data was performed on 14 xenograft passages by bioinformatic methods.

**Results:**

The most frequent losses and gains of DNA copy number were detected at 9p21.3, 16q and at 8, 15, 17q21.32-qter, 1q21.1-qter, respectively. The presence of these alterations was consistent in all tumor passages. aCGH profiles of xenograft passages of each series resembled their corresponding primary tumors (passage 0). MiR-21, miR-31, miR-31*, miR-106b, miR-145, miR-150*, miR-371-5p, miR-557 and miR-598 showed recurrently altered expression. These miRNAS were predicted to regulate many ES-associated genes, such as genes of the IGF1 pathway, *EWSR1, FLI1 *and their fusion gene (*EWS-FLI1*). Twenty differentially expressed miRNAs were pinpointed in regions carrying altered copy numbers.

**Conclusion:**

In the present study, ES xenografts were successfully applied for integrated microarray analyses. Our findings showed expression changes of miRNAs that were predicted to regulate many ES associated genes, such as IGF1 pathway genes, *FLI1, EWSR1*, and the *EWS-FLI1 *fusion genes.

## Background

Due to active international collaboration in the study of rare tumors, such as in Ewing's sarcoma (ES), a great body of tumor-related molecular biomarkers have already been mined by novel array technologies and the clinical significance of some of the biomarkers has been established [1]. A limiting factor for the research of rare bone tumors has been the limited availability of research material derived from patients. Therefore, xenografts, tumors grown from human tumor cells and implanted in immunodeficient animals, are a viable option that is widely used for *in vivo *models [2,3]. Xenografted tumors are enriched for neoplastic cells with the minimal contaminating mouse stromal tissue, a property that makes them suitable for molecular analysis [4]. Several studies have shown that xenograft tumors may provide an accurate reflection of tumor biology [5-9].

MicroRNAs (miRNAs) are small, single-stranded non-coding endogenous RNAs, consisting of 20-23 nucleotides, typically acting as post-transcriptional repressors [10,11]. Despite the fact that miRNAs have been implicated in more than 70 diseases, they have never been investigated, to our knowledge, in the tumor/xenograft setting [12] (http://cmbi.bjmu.edu.cn/hmdd).

Here, we have performed miRNA- and comparative genomic hybridization (CGH) array analyses on a series of ES xenografts to investigate differential miRNA expression and genomic DNA copy number changes, which are potentially involved in the tumorigenesis of ES. These results have been assessed to identify whether copy number alterations influence miRNA expression, since DNA copy number abnormalities can have a direct impact on the miRNA expression levels [13]. Multiple xenograft passages from each primary tumor were tested to enhance the statistical power of the study.

## Methods

### Samples

Originally six xenograft series originating from ES patients (5 primary tumors and a lung metastasis) comprising 34 passages in total were obtained from the Department of Pathology, University of Valencia, Spain. Two series of xenograft passages originated from one patient with both the primary tumor and the metastatic tumor in the lung. Although all of the 34 passages were used in the aCGH study, only 14 out the 34 passages were available for the miRNA study (Table 1). These 14 passages represented original 5 xenograft series, including both early and advanced passages. The passage 0 that represented primary tumor and was available for four series of the xenografts was not, however, available for miRNA profiling. The *EWS-FLI1 *and *EWS-FEV *translocations were present in 4 and 1 of the primary tumors, respectively, and were retained in all xenografts. To select an optimum control for any kind of expression analysis is generally considered a difficult task; we ended up with two human mesenchymal stem cell samples from different cell cultures for use as controls. Mesenchymal stem cells have been utilized as control samples in many previous expression studies due to the convincing evidence that supports the mesenchymal stem cell origin of ES [13-15]. DNA microarray analysis, as well as functional studies, have revealed the relationship between ES and mesenchymal stem cells [16,17] as well as between ES and endothelium, and fetal neural crest [18,19], further sustaining the fact that, despite all the efforts, the origin of ES is still a matter of dispute. Very likely ES derives from much undifferentiated cells. In our analysis, we used mesenchymal stem cell as the calibrator, in analogy to other reports recently published [20,21].

**Table 1:** Ewing sarcoma xenograft series, 6, originating from five patients

Case No. (Nude)	Xenograft Passage
488 (15)	1*, 2*, 4, 7*, 11, 14*
445 (22)	0, 1, 4, 11, 15, 22
451 (53)	0, 4, 11*, 15*, 18, 21*
455 (199)	0, 1, 5*, 11, 17, 25*
430 (PRI) (230)	0, 1*, 4, 9, 19*
430 (MET) (248)	1*, 4, 14*, 21, 30*

Case number 430 has two xenograft passages originating from one patient in different status of tumor: PRI = Primary Tumor, MET = Lung Metastasis. Samples used in the miRNA study are marked with an asterisk. Xenograft passage number 0 refers to the corresponding primary patient sample

The stem cells were obtained from human primary bone marrow-derived mesenchymal stem cells after informed patient consent; precisely, from bone marrow aspirates (iliac crest) of patients undergoing hip replacement surgery. Nucleated cells were placed in modified alpha-MEM media (Li StarFish) containing 20% fetal bovine serum (Cambrex Bioscience), 100 units/mL penicillin (Life Technologies), 100 mg/mL streptomycin (Life Technologies), and 2 mmol/L glutamax (Life Technologies). Confluent cells were harvested by trypsin/EDTA and seeded at 1:3 density.

### Xenografts

Male nude mice were purchased from IFFA-CREDO (Lyon, France) and kept under specific pathogen-free conditions throughout the experiment (with vinyl isolates plus sterilized food, water, cage, and bedding). The specimens for xenografting were obtained from the surgery of original tumors and placed in the culture medium (RPMI 1640) with antibiotics at 37°C until the transplantation (usually less than 2 hours after the surgery). Various fragments of the non-necrotic tumor, about 3-5 mm in size, were xenografted into the subcutaneous tissue of the backs of nude mice. The cells from this first implantation are denoted as passage 0 cells and are considered to represent primary tumors. After allowing the growth to approximately 2-3 cm, the subsequent tumor transfers were performed following the same procedures as in the initial xenotransplant and always under highly sterile conditions. In each passage, sufficient amount of material was obtained for the histopathology analysis (Formalin-fixed paraffin-embedded tissue blocks from which tissue microarrays were constructed), the touch preparations, the electron microscopy, the tissue culture, and frozen tissue. All the experimentation involving laboratory animals was approved by the Institutional Animal Care of Valencia University and the Local Government and was performed in accordance with the national legislation of Spain.

The ploidy analysis was not seen necessary to be performed as both histopathological and copy number analysis did not provide any evidence of polyploidy.

### Nucleic acid isolation

Genomic DNA from the 34 passages (Table 1) was extracted by the standard phenol-chloroform method. Reference DNAs, male and female, were extracted from the pooled blood samples (4 individuals each) obtained from the Blood Service, Red Cross, Finland. The Qiagen's miRNeasy Mini Kit (Qiagen, Valencia, CA, USA) was used to extract total RNA, including miRNA, according to the manufacturer's instructions. The Nanodrop-1000 spectrophotometer (Thermo Fisher Scientific Inc., Wilmington, DE, USA) was used for quantification of DNA and RNA. The quality of DNA was checked by gel electrophoresis, while for the quality of total RNA and miRNA, the Agilent bioanalyzer (Agilent Technologies, Santa Clara, CA, USA) was applied.

### Array CGH hybridization, scanning and data analysis

The Agilent Human Genome CGH 4x44A oligo microarrays (Agilent Technologies, Santa Clara, CA, USA) containing ~44,000 oligonucleotide probes were used. Digestion, labeling, and hybridization of DNA were done according to the manufacturer's instructions (Agilent protocol version 2.0). Briefly, the same amounts (1.5 μg) of patient DNA and gender matched reference DNA were digested. The digested DNAs were labelled by random priming with Cy3-dUTP (reference DNA) and Cy5-dUTP (patient DNA) by use of the Agilent Labelling Kit, after which the labelled DNAs were purified. Next, differentially labelled patient and reference DNAs were combined and hybridized to Agilent Human Genome CGH 4x44A microarrays at 65°C for 24 hours. The hybridized arrays were washed and, then, scanned with the Agilent's microarray scanner (G2565BA, Agilent Technologies, Santa Clara, CA, USA). The raw data was extracted from the array images by the Agilent's Feature Extraction Software (version 8.1). The data was analyzed with the Agilent CGH Analytics software (version 3.4) using ADM-2 algorithm (threshold 6.0) with 1.0 Mb window size.

### MicroRNA hybridization, scanning and data processing

We used the Agilent's miRNA microarray system (V3), containing 866 human and 89 human viral miRNAs catalogued in the Sanger miRNA database v12 (Agilent Technologies, Santa Clara, CA, USA). Labelling and hybridization of RNA samples was performed with the Agilent's miRNA Complete Labelling and Hyb Kit. Accordingly, 100 ng of total RNA were treated with Calf Intestine Phosphatase for 30 min at 37°C; 100% DMSO was used for denaturation at 100°C for 5 min, after which the samples were immediately transferred into an ice water bath to prevent reannealing. Next, samples were labelled with cyanine 3-pCp by incubating with T4 RNA ligase for 2 hours at 16°C. After the labeling reaction, the samples were vacuum dried at medium heat and re-suspended in nuclease-free water. Next, samples were hybridized to the microarrays in the Agilent SureHyb chambers (Agilent Technologies) for 20 hours at 55°C, after which the microarrays were washed with the manufacturer's washing buffers. The arrays were scanned using the Agilent's scanner and the raw data were preprocessed with the Agilent's Feature Extraction Software with default parameters. Details of the miRNA preprocessing protocol are provided by the manufacturer.

Statistical analysis was carried out with the GeneSpring GX analysis software (version 10) and the R statistical programming language (http://www.r-project.org). The data were preprocessed by adding offsets and carrying out normalization between all the arrays by the quantile method, and taking log2 transformation. The data were filtered by removing control miRNAs and the miRNAs that were not detected across any of the samples. Detection calls were provided by the Agilent's Feature Extraction Software. MiRNAs with less than the threshold of the ratio of total gene signal/total gene error under three were considered to be undetected. The detected miRNAs were regarded as present in the measured sample. We also removed miRNAs based on their expression: for each miRNA, its expression had to exceed in at least one array (negative control miRNAs' expression) + 1.5× standard deviation (negative control miRNAs' expression). We examined the detection calls for each sample to determine which miRNAs were expressed or not expressed. To find differentially expressed miRNAs between controls and xenograft samples, we performed moderated t-tests for each miRNA to find significant differences in miRNA expression between the two groups and, then, calculated the most significant up-regulated and downregulated miRNAs (adjusted p value [q value] < 0.05, Bonferroni correction to correct for multiple testing).

### Real-time polymerase chain reaction (real-time RT-PCR) analysis

To validate the selected miRNA expression levels in ES samples compared to control samples, RT-PCR analysis was applied. The miScript Reverse Transcription Kit (Qiagen, Valencia, CA) served for reverse transcription of RNA, according to manufacturer's guidelines. QRT-PCR was performed on a Light-cycler, software v.3.5 (Roche Applied Science, Mannheim, Germany) by the SYBR Green miScript PCR system (Qiagen). Each reaction was performed in a 20-μl volume with 5 ng template cDNA. The primers for amplification of selected miRNAs and snRNA U6 were purchased from the Qiagen. The experiments were performed in duplicate for each RNA sample, and every run included a control without template. The U6 primer assay (Qiagen) served as an endogenous control for normalization. The relative quantification (RQ) for each miRNA, compared with U6 was calculated using equation 2^-ΔΔCt^.

### Relationship between miRNA and CGH data

We investigated whether any association existed between miRNA expression changes and gain/loss of genomic regions. We mapped each miRNA to its chromosomal band location, which was retrieved from the Ensembl, using the biomaRt package, and the mirBase database. For each miRNA, we counted the number of xenograft samples (out of 14) in which there was loss, gain, or no change in copy number for the corresponding chromosomal band. Possible associations were determined by counting the number of samples showing miRNA over-expressed/genomic gain and miRNA under-expressed/genomic loss. We also counted the number of control samples (out of 2) in which the miRNA was detected.

### Predicted targets of differentially expressed miRNAs

After having acquired the differentially expressed miRNAs, we used the miRBase target prediction database (http://microrna.sanger.ac.uk), TargetScan (http://www.targetscan.org), and miRanda (http://www.microRNA.org) for evaluation of the predicted mRNA targets. The list of predicted mRNA targets was screened for the genes known to be functionally relevant in ES and predicted at least by one of the algorithms.

## Results

### Copy number alterations in xenografts

**Figure 1:**
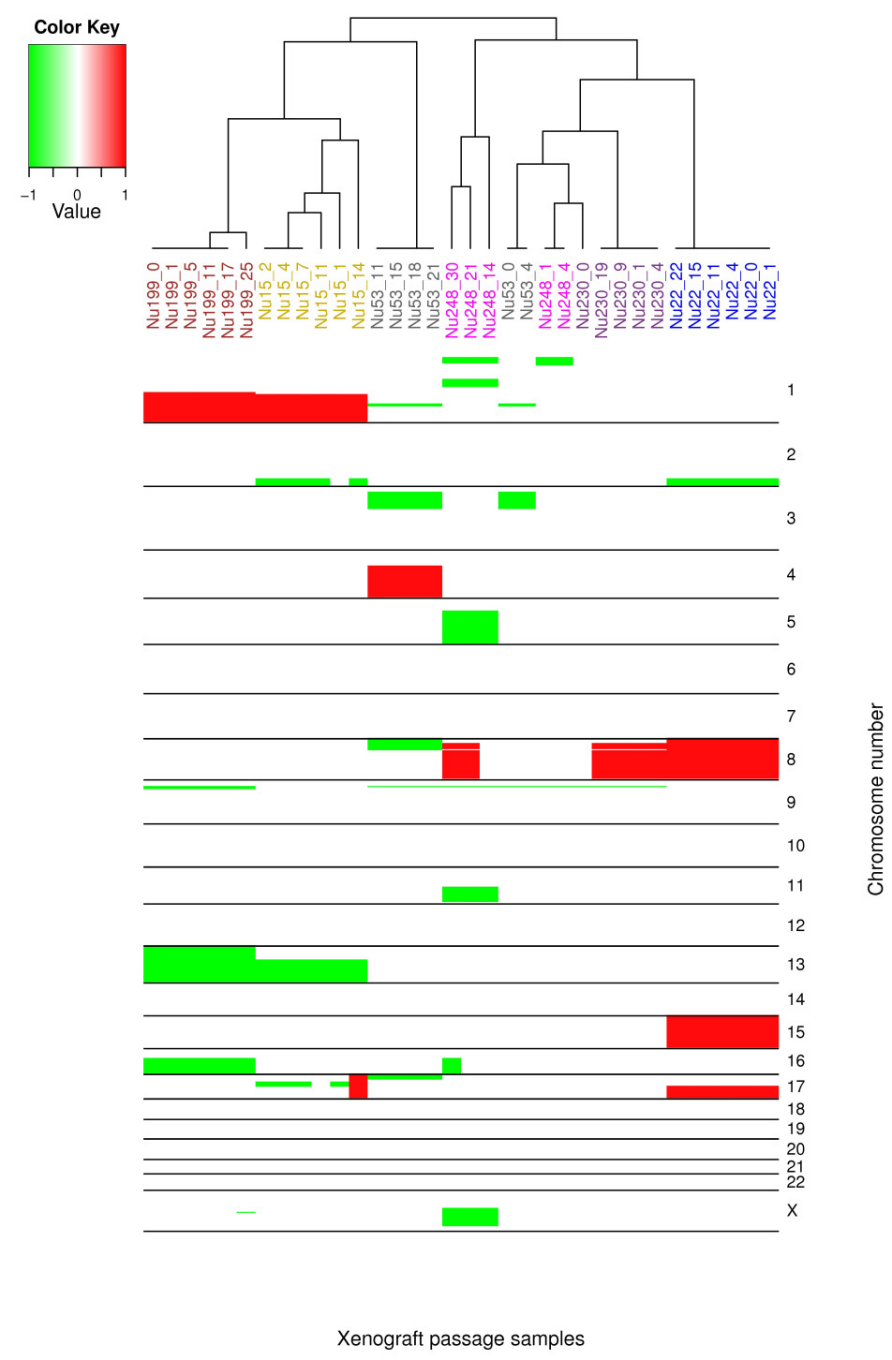
**Copy number changes on each chromosome were ordered using hierarchical clustering**. Most of the xenograft passages of each series clustered together and also with the passage 0, its corresponding primary tumor.

By the aCGH analysis, xenograft passages displayed a total of 28 copy number changes, of which approximately half appeared in every passage of each series whilst the other half were present in some of the passages of each series (Table 2, and 3). All these changes were evident in passage 0. Moreover, the clustering analysis of aCGH profiles for each cytogenetic location indicated that the aCGH profiles of the passages 0 as primary tumors and the rest of the xenograft passages were similar (Figure 1). Copy number losses (65%) were more frequent than gains (35%). The most frequent copy number losses were seen at chromosomal regions 9p21.3 and 16q; these were observed in four (63%) and two (20%) series of xenografts passages, respectively. The frequent gain at whole chromosomes 8 and 15 (17%) occurring together with a gain at 17q21.32-qter (17%) was observable in all passages of a series (case number 445). The genomic region 1q21.1-qter frequently displayed gain. Changes in copy number were acquired during the growth period of xenografts including gains at 2q35-q37.3, 4q13.3-qter, 8p11.21-p21.2 and 8q and losses at 8p, 17p, 13, Xq21.1, 1p13.3-p31.1, 5q, 11q13.4-q24.3, Xq12-q26.3 and 16q. In one xenograft series (Case number 488), loss of 17q12-q21.32, that was present in the early passages, disappeared during the growth process. The loss of 1p36.12-pter in the first two passages originating from lung metastasis (1 and 4) changed to loss of 1p36.21-pter in the last three passages (14, 21, and 30). The lung metastasis xenografts showed 9 copy number changes, whereas only 3 of these aberrations were observable in the xenograft passages from its primary tumor.

**Table 2:** The copy number changes present in all the passages of each xenograft series

Case No. (Nude)	Array CGH results
488 (15)	+1q21.1-qter, -13q14.12-qter
445 (22)	-2q35-q37.3 (uncontinuous), + 8, +15, +17q21.32-qter
451 (53)	-1q24.3-q25.2, - 3p12.3-p24.3, -9p21.3
455 (199)	+1q, -16q, -9p21.3
430 (PRI) (230)	-9p21.3
430 (MET) (248)	-1p36.12-pter, -9p21.3

PRI = Primary Tumor, MET = Lung Metastasis

**Table 3:** Copy number changes present in only part of the passages of each xenograft series

Case	Nude- Passage	Array CGH result
488	15- 2, 4, 7, 11, 14	-2q35-q37.3
488	15- 1, 2, 4, 7	-17q12-q21.32
488	15- 14	+17
451	53- 11, 15,18, 21	+4q13.3-qter, -17p
455	199- 5, 11, 17, 25	-13
455	199- 25	-Xq21.1
430 (PRI)	230- 1, 4, 9, 19	+8p11.21-p21.2, +8q
430 (MET)	248- 1, 4	-1p36.12-pter
430 (MET)	248- 14, 21, 30	-1p36.21-pter, -1p13.3-p31.1, -5q,
		-11q13.4-q24.3, -Xq12-q26.3
430 (MET)	248- 21, 30	+8p11.21-p21.2, +8q
430 (MET)	248- 30	-16q

PRI = Primary Tumor, MET = Lung Metastasis

### MicroRNA alterations in xenografts

**Figure 2:**
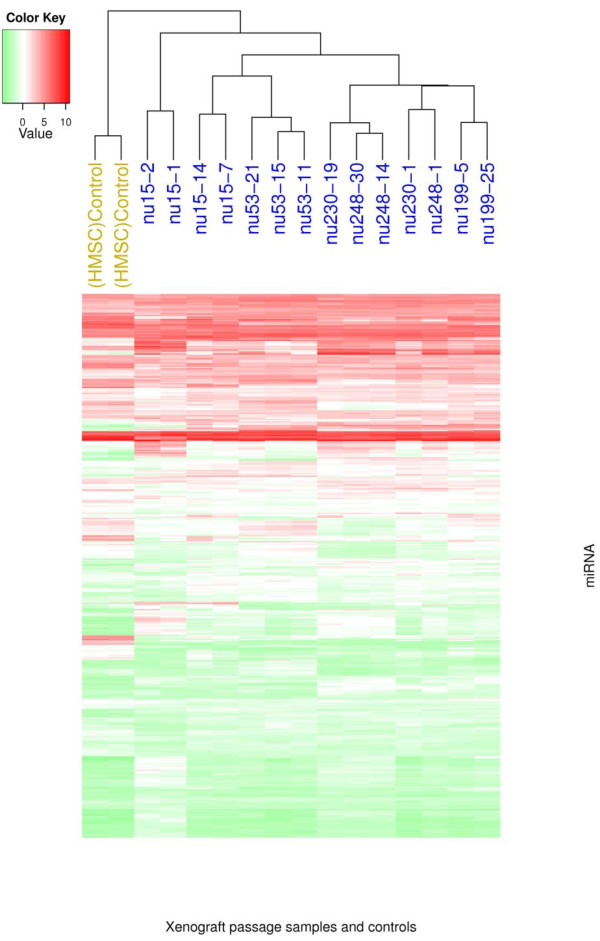
**This picture shows the miRNAs detected in metastasis and corresponding primary tumor xenograft passages and control samples**. In common, these three sample types comprised 191 miRNAs. In addition to these, 98 miRNAs were expressed in both the metastasis and the corresponding primary tumor xenograft passages, 22 miRNAs were exclusively expressed in metastatic xenograft passages, 12 miRNAs were exclusive to xenografts from primary tumor, and 11 miRNAs were expressed as well in controls as in primary tumor xenograft passages.

Differences in miRNA expression between xenografts and control samples were detected upon analysis (Figure 2). Exclusively expressed miRNAs were detected; two in control samples (miR-31, miR-31*) and 46 in all xenograft passages (Table 4). In addition, 5 miRNAs (miR-106b, miR-93, miR-181b, miR-101, miR-30b) were significantly over-expressed (q-value < 0.05), while 6 miRNAs (miR-145, miR-193a-3p, miR-100, miR-22, miR-21, miR-574-3p) were significantly under-expressed across the xenograft passages in relation to the controls (q-value < 0.05). Xenografts from primary and control samples were compared to xenograft passages from the lung metastasis (Case number 430), to determine differences in miRNA expression. A set of 30 miRNAs were found to have differential expression; 18 exclusively expressed in lung metastatics xenografts and 12 in primary tumor xenografts.

**Table 4:** The 46 miRNAs detected in all xenografts samples, while absent from all control samples.

miRNA	miRNA	miRNA	miRNA
hsa-miR-1224-5p	hsa-miR-451	hsa-miR-188-5p	hsa-miR-629*
hsa-miR-126*	hsa-miR-483-5p	hsa-miR-652	hsa-miR-663
hsa-miR-1290	hsa-miR-486-5p	hsa-miR-19b-1*	hsa-miR-7-1*
hsa-miR-1300	hsa-miR-194	hsa-miR-215	hsa-miR-744
hsa-miR-135a*	hsa-miR-195*	hsa-miR-219-5p	hsa-miR-877*
hsa-miR-142-3p	hsa-miR-501-3p	hsa-miR-873	hsa-miR-9
hsa-miR-144	hsa-miR-502-3p	hsa-miR-30c-1*	hsa-miR-9*
hsa-miR-150	hsa-miR-505*	hsa-miR-328	
hsa-miR-150*	hsa-miR-223	hsa-miR-338-3p	
hsa-miR-181c*	hsa-miR-564	hsa-miR-371-5p	
hsa-miR-548c-5p	hsa-miR-421	hsa-miR-345	
hsa-miR-557	hsa-miR-339-3p	hsa-miR-378	
hsa-miR-33a	hsa-miR-598	hsa-miR-629	

**Figure 3:**
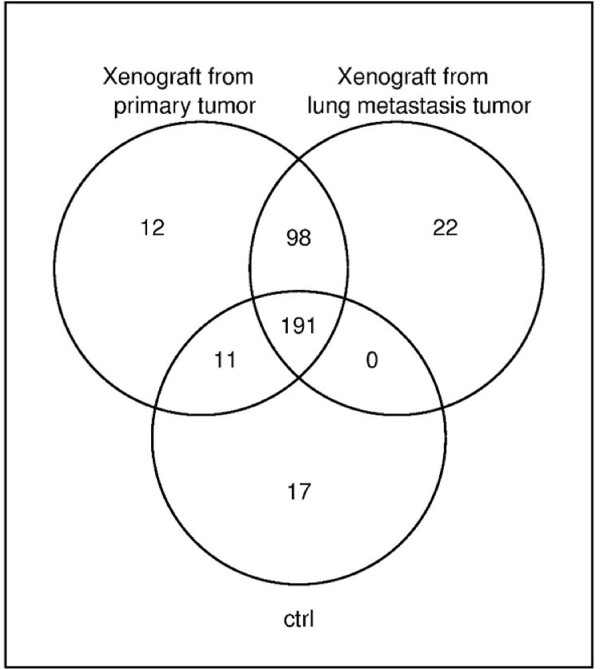
**Hierarchical clustering of the xenograft passages**. Note that the xenograft passages show a distinct expression profile that separates them from the mesenchymal stem cell control samples.

Eleven miRNAs were expressed in both control samples and primary tumor xenograft passages but not at all in metastatic samples (Table 5, Figure 3). Nine of these (miR-214*, miR-154*, miR-337-3P, miR-369-5p, miR-409-5p, miR-411, miR-485-3p, miR-487a, miR-770-5p) were also preferentially expressed in other primary tumor xenografts when compared to metastatic xenograft passages.

**Table 5:** MiRNAs expressed in xenograft passages of A) Case 430 primary tumor while absent in lung metastasis, 12 miRNAs, B) Case 430 lung metastasis while absent in primary tumor, 18 miRNAs and C) Case 430 primary tumors and control, while absent in lung metastasis, 11 miRNAs

miRNAs expressed in			
A) Xenograft passages from Primary tumor (12 miRNAs)	B) Xenograft passages from lung metastasis (18 miRNAs)		C) Control and xenograft passages from Primary tumor (11 miRNAs)
hsa-miR-1237	hsa-miR-1183	hsa-miR-595	hsa-miR-154*
hsa-miR-139-3p	hsa-miR-124	hsa-miR-601	hsa-miR-214*
hsa-miR-139-5p	hsa-miR-1471	hsa-miR-623	hsa-miR-337-3p
hsa-miR-202	hsa-miR-32*	hsa-miR-662	hsa-miR-34a*
hsa-miR-30b*	hsa-miR-424*	hsa-miR-664*	hsa-miR-369-5p
hsa-miR-450a	hsa-miR-486-3p	hsa-miR-671-5p	hsa-miR-409-5p
hsa-miR-490-3p	hsa-miR-520b		hsa-miR-411
hsa-miR-501-5p	hsa-miR-520e		hsa-miR-485-3p
hsa-miR-502-5p	hsa-miR-96		hsa-miR-487a
hsa-miR-548 d-5p	hsa-miR-877		hsa-miR-542-3p
hsa-miR-602	hsa-miR-95		hsa-miR-770-5p
hsa-miR-885-5p	hsa-miR-765		

### Validation of differentially expressed miRNAs by qRT-PCR

QRT-PCR was used to validate the expression levels of four selected miRNAs that showed altered expression in the miRNA microarray results. The selection of miRNAs for further validation was based on the expression level of miRNA microarray results and on the level of representation in the expression categories observed (i.e. exclusively expressed, significantly under-expressed and significantly over-expressed). The miR-31 and miR-31* were exclusively expressed in control samples and absent in xenograft passages, while miR-106b was significantly over-expressed and miR-145 significantly under-expressed, respectively, in xenograft samples compared to control samples.

As for the validation results by qRT-PCR, the expression levels of miR-31, miR-31* and miR-145 were under-expressed in the xenograft samples compared to the control samples (relative expression 0.00062, 0.00809 and 0.09111, respectively). These results are consistent with the miRNA microarray results. Similarly, the over-expression of miR-106b in xenograft samples seen in miRNA microarray was confirmed by qRT-PCR results showing relative expression level of 87.7.

### Relationship between miRNAs and copy number alterations

A joint analysis of the aCGH data and miRNA data for the 14 xenograft passages, which were common to both studies, was performed by looking for miRNAs whose expression was correlated with a change (loss/gain) at their chromosomal location. Three criteria were used to determine the miRNAs of greatest interest: (i) differentially expressed miRNAs in all 14 xenograft passages, (ii) altered miRNAs whose chromosomal locations were affected by the same copy number changes in most of the passages, and (iii) miRNAs fulfilling both previous criteria.

Of the 46 miRNAs exclusively expressed in all xenograft passages, 7 miRNAs (miR-144, miR-195*, miR-215, miR-451, miR-454, miR-557, miR-744) were located in chromosomal regions with a copy number gain in at least one of the passages. Four miRNAs that displayed absent or severely reduced expression in any xenograft passages (miR-22, miR-31, miR-31*, miR-145) were located in chromosomal regions with a copy number loss in at least 2 of the passages. In addition, five passages displayed gains of a chromosomal region that contained 3 frequently expressed miRNAs (miR-765, miR-135b and miR-29c*); miR-765 and miR-135b were expressed in 10 passages while miR-29c* was expressed in 12 passages but in none of the control samples (Table 6).

**Table 6:** Altered miRNAs in regions of copy number changes

miRNA in copy number gain			miRNA in copy number loss		
	**Chr**.	Number of samples		**Chr**.	Number of samples
miRNA	location	in gain region	miRNA	location	in loss region
miR-765	1q23.1	5	miR-137	1p21.3	2
miR-135b	1q32.1	5	miR-143*	5q32	2
miR-29c*	1q32.2	5	miR-143*	5q32	2
miR-557	1q24.2	6	miR-145*	5q32	2
miR-215	1q41	6	miR-145	5q32	2
miR-744	17p12	1	miR-31	9p21.3	10
miR-195*	17p13.1	1	miR-31*	9p21.3	10
miR-451	17q11.2	1	miR-22	17p13.3	3
miR-144	17q11.2	1	miR-22*	17p13.3	3
miR-454	17q22	1	miR-503	xq26.3	2

Chr. = Chromosome

## Discussion

Here we have sought to identify differentially expressed miRNAs in ES xenografts and to investigate the underlying molecular changes by integration of these results with aCGH analysis of the same samples.

### MiRNA expression profile of ES xenografts

Xenografts displayed 60 differentially expressed miRNAs that distinguished them from control samples (Human mesenchymal stem cells). Of these, 46 miRNAs were exclusively expressed in xenografts while 2 (miR-31 and miR-31*) miRNAs were exclusively expressed in controls. The remaining 5 miRNAs (miR-106b, miR-93, miR-181b, miR-101, miR-30b) were significantly over-expressed while 6 miRNAs (miR-145, miR-193a-3p, miR-100, miR-22, miR-21, miR-574-3p) were significantly under-expressed in xenografts. The expression profiles of 4 miRNAs (miR-31, miR-31*, miR-106b, miR-145) were confirmed by RT-PCR.

To evaluate the potential role of the differentially expressed miRNAs, three databases were searched for the known ES-associated genes targeted by these miRNAs, by applying target prediction algorithms. The targets included *EWSR1 *(GeneID: 2130), *FLI1 *(GeneID: 2313), *SOX2 *(GeneID: 6657), *p53 *(GeneID: 7157), *IGFBP3 *(GeneID: 3486), *IGF1 *(GeneID: 3479) and *IGF1R *(GeneID: 3480). The differential expression of the miRNAs regulating these genes may play a role in the tumorigenesis and tumor progression of ES.

Interestingly, miR-150, which targets the tumor suppressor gene *TP53*, was expressed in all xenograft samples but in none of the control samples. This is in accordance with the study of Fabbri and colleagues [22] who have included TSGs in their investigation of likely over-expressed miRNA target genes. In addition, one of our xenograft series (Case number 451) showed losses at 17p, containing *TP53*, that appeared in later passages. Previous ES studies have shown that, despite the low frequency of mutations in *TP53*, an alteration of *TP53*, in conjunction with the deletion of *CDKN2A*, is associated with a poor clinical outcome [23,24]. Moreover, the homozygous deletion of this gene has been reported in a small subset of ES patients [25,26].

The IGF-1 pathway, whose genes *IGF1R, IGF-1 *and *IGFBP-3 *are among the target genes of the differentially expressed miRNAs, plays a critical role in cancer development, including ES [26-28]. *IGF1R *is targeted by miR-145 and miR-31*, and previous studies have shown*IGF1R *to be a direct target of miR-145 [29] as well as to be over-expressed in Ewing tumors [27,28]. As for *IGF-1*, it is the target of 11 miRNAs including miR-21, miR-31, miR-145, miR-150, miR-194, miR-215, miR-421, miR-486-5p, 548c-5p, and miR-873. Interestingly, *IGFBP3*, which is among the target genes of miR-150*, was, in our study, expressed in all xenografts but not in control samples. *IGFBP-3*, which is a major regulator of cell proliferation and apoptosis, inhibits the interaction of IGF-1 with its receptor (IGF1R) [30-33]. Indeed, it has been reported that high IGF-1 and low IGFBP-3 levels in serum increase the risk of cancer [26]. IGFBP3 is strongly down-regulated by the *EWS/FLI-1 *fusion gene [34], which is able to induce expression of embryonic stem cell gene *SOX2*. Consequently, *SOX2 *participates in ES cell proliferation and tumorigenesis and might play a central role in ES pathogenesis [35]. As for our study, *SOX2 *was among the target genes of miRNA-21 that showed under-expression in xenografts. Another under-expressed miRNA, miR-145, was previously found to target *FLI1 *and its increased expression leads to a decreased migration of microvascular cells in response to the growth factor gradients *in vitro *[36].

Finally, miR-106b targets *EWSR1*, which undergoes a chromosomal translocation to produce the *EWS-FLI *fusion gene in a majority of ES cases, where it is commonly considered to trigger the condition. The action of miR-106b is, thus, likely to only impact on the original/unmodified locus for *EWSRI *since the *EWS-FLI *lacks the 3' portion of *EWSR1*. Further studies would, naturally, be required to confirm this hypothesis.

The alteration of 41 miRNAs was observed in xenograft passages derived from lung metastatic, which may play a crucial role in triggering tumor metastasis. Eight of these miRNAs, all located at the 14q32 imprinted domain (miR-154*, miR-337-3P, miR-369-5p, miR-409-5p, miR-411, miR-485-3p, miR-487a, miR-770-5p) were not expressed in metastasis xenografts but in control samples, thus suggesting a tumor suppressor function. Interestingly, gastrointestinal stromal tumors (GISTs) have displayed 44 expressed miRNAs originatingfrom the 14q32 chromosomal region, for which the low expression of miRNAs was related to tumor progression [37]. A report by Saito and colleagues [38] suggests that miRNAs located in this region function as tumor repressor genes and changes in the methylation status of their promoters could trigger cancer development. This evidence suggests that the miRNAs identified in our study may act as tumor repressors and their absence could increase the risk of metastasis and tumor progression in ES.

### Copy number aberrations in ES xenografts

The most recurrent copy number alterations detected in our CGH analysis (gains at chromosome 8, 1q and losses at 9p21.3 and 16q) are in agreement with other findings on ES patients [1,39-46]. The crucial role of these changes, gains in 1q, 8 and losses of 9p21.3 (including loss of *CDKN2A*) and 16q, has been clarified by notable tumor development and adverse clinical outcome [42,47,48]. These copy number changes were seen throughout the whole xenograft series. In all passages of lung metastasis, losses were observed at 1p36.12-pter/1p36.21-pter. Of note, deletion of this site (1p36) has been found to be related to a poor clinical outcome in ES[43,47]. The loss of 1p36.12-pter in the first two passages originating from lung metastasis (1 and 4) changed to loss of 1p36.21-pter in the last three passages (14, 21 and 30). The lung metastasis xenografts showed 9 copy number changes, whereas only 3 of these aberrations were observable in the xenograft passages from its primary tumor. Likewise in many tumors during the disease progression, the increase of genomic instability is also seen here. This instability most probably explains the variation of the size of 1p deletion. The fact that the terminal part is retained in the deletion emphasizes the importance of 1p36.21-pter region in the selection and in the disease progression.

Somatic mosaicism/heterogeneity occurs commonly in tumors and plays an important role in the progression of the tumor and, thereby, can also explain why some xenograft passages show copy number changes and others do not.

### Integration of miRNA expression profiles and DNA copy number changes

DNA copy number abnormalities can have a direct impact on miRNA expression [49]. In the current study, 20 differentially expressed miRNAs were located in the copy number altered regions. These findings are in accordance with Calin et al. (2004) who observed that half of the miRNAs are located in cancer-associated regions of chromosomes as well as in genomic regions frequently amplified or lost in cancer [49]. The target genes for many of the changes are still unknown and, therefore, miRNAs could well be considered to be the drivers of the underlying changes.

MiR-31 and miR-31*, targeting *IGF1 *and *IGF1R*, are located at the frequently deleted region of 9p21.3, containing the *CDKN2A *gene, which was frequently lost in our samples. Under-expression of miR-31 or deletion of the miR-31 genomic locus is also found in human breast cancers. This miRNA regulates metastasis by opposing local invasion and metastatic colonization in this cancer [50-52]. Chromosome 1 gain is a frequent gain that contains the whole chromosome and seems to be poor prognostic sign [53]. Interestingly, in our study five overexpressed miRNAs (miR-765, miR-135b, miR29c, miR-215, and miR-557) (Table 6) were associated to 1q gain. These candidate miRNAs have an important role and could explain the underlying mechanism in ES. Nevertheless, functional validations of the predicted target genes are needed to better understand their role in the ES tumorgnesis.

## Conclusions

The current study provides new information about the miRNA expression and its relationship with the associated genomic copy number changes in ES xenografts. Our findings suggest that miRNAs play a role in the molecular pathogenesis and tumorigenesis of ES by regulating important genes in the IGF1 pathway as well as the genes *FLI1, EWSR1*, and the *EWS-FLI1 *fusion gene. In addition, integration of the data for gene copy number changes and miRNA profiles demonstrated some cases where the differential expression of miRNAs was the result of copy number alteration of the miRNA genomic locus. Moreover, our study showed that the xenografts can reflect well the genomic pattern of their original tumor.

## Competing interests

The authors declare that they have no competing interests.

## Authors' contributions

NM and MG have equally contributed to this study. SK, as a senior researcher, designed the study and participated in writing the manuscript. NM performed the laboratory work and participated in writing. SS and TN performed the array CGH analysis and contributed to the design of the study plan. MG participated in writing. GL participated in designing the statistical analysis and preparing the manuscript. EE, US-P, M-LK-J and AR participated in designing the study and provided clinical data. All authors contributed to the manuscript and approved the final version of it.
